# An improved method for estimating low LDL-C based on the enhanced Sampson-NIH equation

**DOI:** 10.1186/s12944-024-02018-y

**Published:** 2024-02-08

**Authors:** Tatiana C. Coverdell, Maureen Sampson, Rafael Zubirán, Anna Wolska, Leslie J. Donato, Jeff W. Meeusen, Allan S. Jaffe, Alan T. Remaley

**Affiliations:** 1 Clinical Center, Department of Laboratory Medicine, National Institutes of Health, Bethesda, MD USA; 2 Lipoprotein Metabolism Laboratory, Translational Vascular Medicine Branch, National Heart, Lung, and Blood Institute, National Institutes of Health, Bethesda, MD USA; 3 Department of Laboratory Medicine and Pathology, Mayo Clinic, Rochester, MN USA; 4 Cardiovascular Laboratory Medicine, Mayo Clinic, Rochester, MN USA; 5 Division of Clinical Core Laboratory Services, Mayo Clinic, Rochester, MN USA

**Keywords:** Cholesterol, Triglycerides, Low-density lipoproteins, Cardiovascular disease, Biomarkers

## Abstract

**Background:**

The accurate measurement of Low-density lipoprotein cholesterol (LDL-C) is critical in the decision to utilize the new lipid-lowering therapies like PCSK9-inhibitors (PCSK9i) for high-risk cardiovascular disease patients that do not achieve sufficiently low LDL-C on statin therapy.

**Objective:**

To improve the estimation of low LDL-C by developing a new equation that includes apolipoprotein B (apoB) as an independent variable, along with the standard lipid panel test results.

**Methods:**

Using β-quantification (*BQ*) as the reference method, which was performed on a large dyslipidemic population (*N* = 24,406), the following enhanced Sampson-NIH equation (*eS* LDL-C) was developed by least-square regression analysis:

$$eS\,LDL-C= \frac{TC}{1.15}-\frac{HDL-C}{1.25}-\frac{TG}{6.99}-\frac{\left(TG\times NonHDL-C\right)}{1120}+\frac{{TG}^{2}}{8910}+\frac{\left(TG\times ApoB\right)}{1240}+\frac{ApoB}{4.54}-4.73$$

**Results:**

The *eS* LDL-C equation was the most accurate equation for a broad range of LDL-C values based on regression related parameters and the mean absolute difference (mg/dL) from the *BQ* reference method (*eS* LDL-C: 4.51, Sampson-NIH equation [*S* LDL-C]: 6.07; extended Martin equation [*eM* LDL-C]: 6.64; Friedewald equation [*F* LDL-C]: 8.3). It also had the best area-under-the-curve accuracy score by Regression Error Characteristic plots for LDL-C < 100 mg/dL (*eS* LDL-C: 0.953; *S* LDL-C: 0.920; *eM* LDL-C: 0.915; *F* LDL-C: 0.874) and was the best equation for categorizing patients as being below or above the 70 mg/dL LDL-C treatment threshold for adding new lipid-lowering drugs by kappa score analysis when compared to *BQ* LDL-C for TG < 800 mg/dL (*eS* LDL-C: 0.870 (0.853–0.887); *S* LDL-C:0.763 (0.749–0.776); *eM* LDL-C:0.706 (0.690–0.722); *F* LDL-C:0.687 (0.672–0.701). Approximately a third of patients with an *F* LDL-C < 70 mg/dL had falsely low test results, but about 80% were correctly reclassified as higher (≥ 70 mg/dL) by the *eS* LDL-C equation, making them potentially eligible for PCSK9i treatment. The *M* LDL-C and *S* LDL-C equations had less false low results below 70 mg/dL than the *F* LDL-C equation but reclassification by the *eS* LDL-C equation still also increased the net number of patients correctly classified.

**Conclusions:**

The use of the *eS* LDL-C equation as a confirmatory test improves the identification of high-risk cardiovascular disease patients, who could benefit from new lipid-lowering therapies but have falsely low LDL-C, as determined by the standard LDL-C equations used in current practice.

**Supplementary Information:**

The online version contains supplementary material available at 10.1186/s12944-024-02018-y.

## Background

Cholesterol carried by low-density lipoproteins (LDL-C) is a key risk marker for Atherosclerotic Cardiovascular Disease (ASCVD) [1] and is commonly calculated based on test results from the standard lipid panel (total cholesterol [TC], high-density lipoprotein-cholesterol [HDL-C] and triglycerides [TG]) [2]. Until recently, LDL-C was almost exclusively calculated by clinical laboratories with the Friedewald equation (*F* LDL-C) [3]. The premise of this calculation method is that in plasma from fasting patients, only three types of lipoprotein particles transport cholesterol, namely LDL, HDL and very low-density lipoproteins (VLDL) [4]. A key part of the equation is the estimation of VLDL-C, which is done by dividing the concentration of TG by 5 when in mg/dL units. To calculate LDL-C, one then simply subtracts the cholesterol that is on HDL and VLDL from TC (LDL-C = TC – HDL-C – TG/5). By using this formula, it avoids the need for the separation of lipoproteins by ultracentrifugation and allowed for the first time the routine reporting of LDL-C by clinical laboratories [3].

In 2013, a more accurate equation called the Martin-Hopkins equation (*M* LDL-C) was developed [5]. This equation is nearly identical to the *F* LDL-C equation, but it uses a series of variable factors instead of the fixed factor of 5 as the TG denominator for estimating VLDL-C. This series of factors can be found in a 180-cell table that is grouped by different TG and non-HDL-C intervals. These factors were empirically determined based on the Vertical Auto Profile (VAP) ultracentrifugation method [6]. The extended Martin-Hopkins equation (*eM* LDL-C) uses an additional set of factors for samples with a TG between 400 to 800 mg/dL [5, 7].

In 2020, a bivariate quadratic equation that depends upon non-HDL-C and TG was described for estimating VLDL-C, which became part of what is known as the Sampson-NIH equation for LDL-C (*S* LDL-C) [8]. Compared to all other equations, LDL-C calculated by this method, particularly for hypertriglyceridemic samples, matched the closest to the β-quantitation (*BQ*) reference method [9, 10], which is used for the standardization of routine diagnostic assays for LDL-C.

Besides the ratio of cholesterol to TG, lipoprotein particle number and their sizes are other important determinants for the cholesterol carrying capacity of lipoproteins. Apolipoprotein B (apoB), the main structural protein on LDL and VLDL, is present as a single copy per lipoprotein particle, and hence it can be used to estimate the total number of apoB-containing lipoprotein [11]. We recently included apoB as an independent variable for improving the estimation of VLDL-C in order to diagnose Type III dysbetalipoproteinemia [12], which is characterized as having cholesterol enriched VLDL particles [13].

In this study, we examined whether we could also improve the accuracy for estimating low LDL-C with the use of apoB as an independent variable. The clinical rationale for developing a new equation is that with the use of the new more effective lipid-lowering drug therapies, it is becoming more common to see patients with extremely low levels of LDL-C. In addition, US guidelines recommend that for the secondary prevention of ASCVD, patients should be treated with a proprotein convertase subtilisin/kexin type inhibitor (PCSK9i) and or another type of lipid-lowering therapy in conjunction with a statin in order to reach an LDL-C below at least 70 mg/dL [1]. For some high-risk patients, even lower LDL-C target goals have recently been recommended by some guidelines [14, 15]. The relatively high cost of PCSK9i therapy, however, can create a barrier to reimbursement by insurance companies [16]. The negative bias of the *F* LDL-C equation, particularly for hypertriglyceridemic patients, can lead to falsely low results below the 70 mg/dL target treatment threshold, which can mislead healthcare providers on the eligibility of patients for PCSK9i therapy. Although the newer LDL-C equations are more accurate than the *F* LDL-C equation, they are still not as widely used and still have less than ideal accuracy at low LDL-C values [9]. The direct measurement of LDL-C by homogenous assays may be useful for patients with low LDL-C, but they are frequently not offered by clinical laboratories and or can have their own analytical challenges [17]. Therefore, we investigated here whether measuring apoB and including it in a new equation called the enhanced Sampson-NIH (*eS* LDL-C) equation can improve the estimation of low LDL-C, particularly for high-risk patients who are possibly candidates for new lipid-lowering therapies.

## Methods

Deidentified lipid and apoB test results from patients for whom the tests were ordered for routine medical care from the Mayo Clinic were used for analysis as previously described [18, 19]. LDL-C and other lipid tests were determined by the *BQ* reference method (*N* = 39,874) [8]. ApoB was measured in a subset of this population (*N* = 24,406), using an immunoturbidometric assay on a Cobas c501 analyzer (Roche Diagnostics, IN). Research under this study was considered non-human subject research and exempted from IRB review.

The e*S* LDL-C equation was established by least square regression analysis on a randomized training dataset of *BQ* LDL-C test results (*N* = 12,196) and then tested on a separate validation dataset of *BQ* LDL-C results (*N* = 12,210). The minimum and maximum of lipid values for the *BQ* LDL-C training dataset are as follows: (HDL-C: 2–201 mg/dL, TC: 27–811 mg/dL, TG: 5–1471 mg/dL, nonHDL-C: 12–777 mg/dL, BQ LDL-C: 9–593 mg/dL, and apoB: 5–401 m/dL)). Regression Error Characteristic analysis was performed as previously described [20]. The agreement between the various LDL-C equations with BQ LDL-C for classifying patients above and below 70 mg/dL was assessed by the calculation of Kappa scores [21]. LDL-C was calculated by the various equations as per their original descriptions [3, 7, 8].

All data analysis was done with JMP software (JMP, Cary, NC) or by Excel (Microsoft, Redmond, WA). Data for key findings and a spreadsheet for performing the new *eS* LDL-C calculation can be downloaded at the NHLBI Fig Share website by searching under the name Sampson.

## Results

**Fig. 1:**
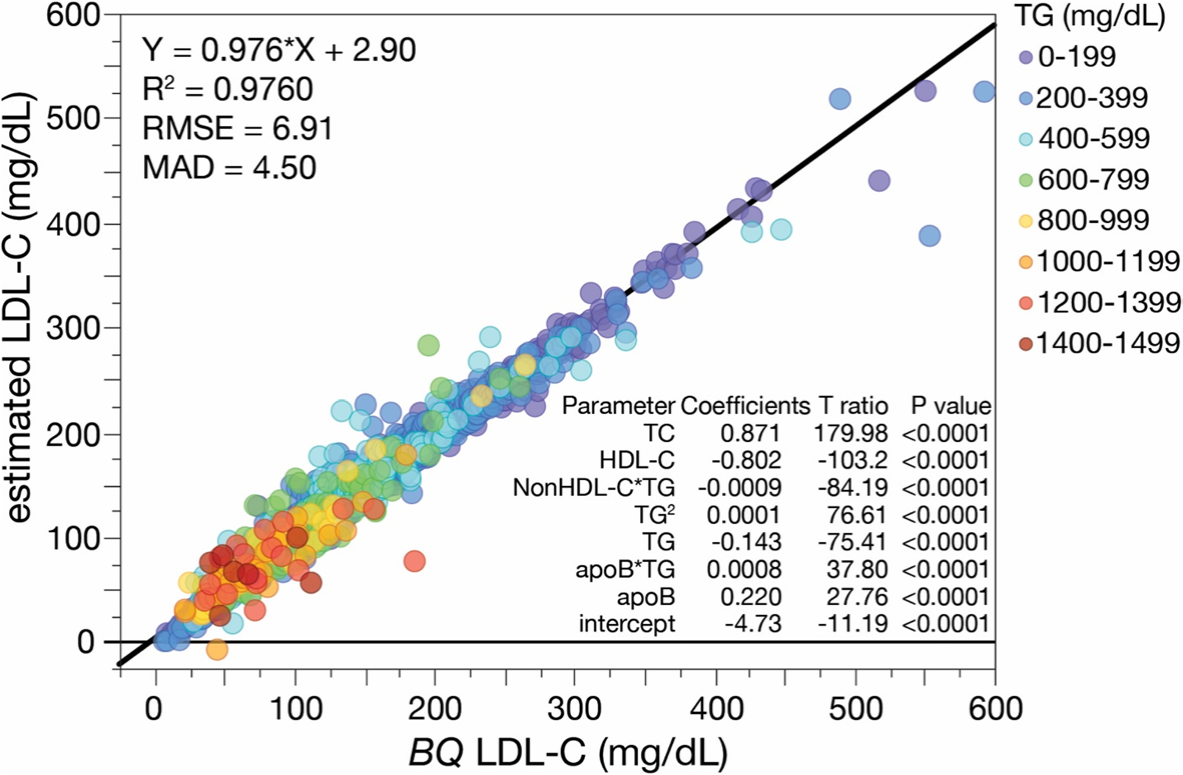
Development of the *eS* LDL-C equation. With LDL-C as measured by *BQ* reference method (*BQ*-LDL-C) as the independent variable, the e*S* LDL-C equation was established by least-square regression analysis on a training dataset (*N* = 12,196). The solid black line is the linear fit for the regression equation. Regression equation and its coefficients are shown in figure. Results are color coded by TG level with the values indicated in the legend

The new *eS* LDL-C equation was established by least squares regression analysis (Fig. 1), using *BQ* LDL-C as the reference method. As can be seen below, the new equation contains all the same individual variables based on the standard lipid panel like the original Sampson equation but the coefficients for the variables differ. It also contains a new variable for apoB and a new interaction term between apoB and TG.

$$eS\,LDL-C= \frac{TC}{1.15}-\frac{HDL-C}{1.25}-\frac{TG}{6.99}-\frac{\left(TG\times NonHDL-C\right)}{1120}+\frac{{TG}^{2}}{8910}+\frac{\left(TG\times ApoB\right)}{1240}+\frac{ApoB}{4.54}-4.73$$

**Fig. 2:**
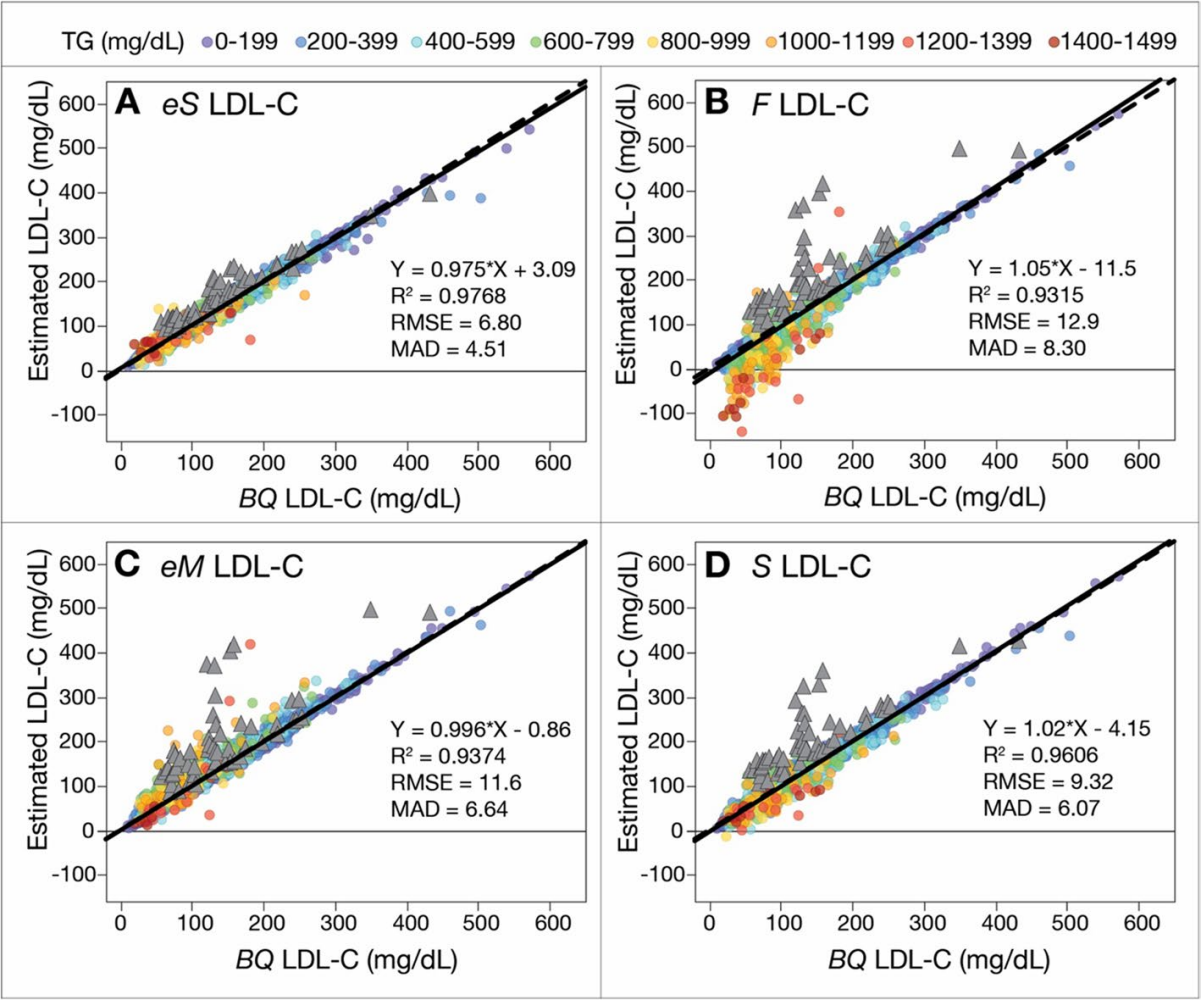
Comparison of estimated LDL-C versus *BQ*-LDL-C. LDL-C was calculated in patients (validation set, *N* = 12,210) with a wide range of LDL-C values by *eS-*LDL-C (**A**), *F*-LDL-C (**B**), *eM*-LDL-C (**C**), and *S-*LDL-C (**D**) equations and plotted against LDL-C as measured by *BQ* reference method (*BQ*-LDL-C). Solid lines are the linear fit for indicated regression equations. Results are color coded by TG level with the values indicated in the legend (mg/dL). Grey triangles are patients with Type III hyperlipidemia

When the *eS* LDL-C equation was applied to the validation dataset (Fig. 2A), it showed similar accuracy based on standard regression parameters (correlation coefficient [R^2^], root mean square error [RMSE], and mean absolute difference [MAD]) as the training dataset, indicating that the new equation was not overfitted. Notably, it also matched much better to the *BQ* LDL-C reference method than the original *S* LDL-C equation or when compared to the *eM* LDL-C or *F* LDL-C equations (Fig. 2B-D). Unlike the *F* LDL-C equation, it did not result in any nonsensical negative LDL-C values for high TG samples. In addition, LDL-C from patients with Type III dysbetalipoproteinemia (gray triangle symbols), which showed a clear positive bias for the three other equations, appeared closer to the regression line for the *eS* LDL-C equation.

**Fig. 3:**
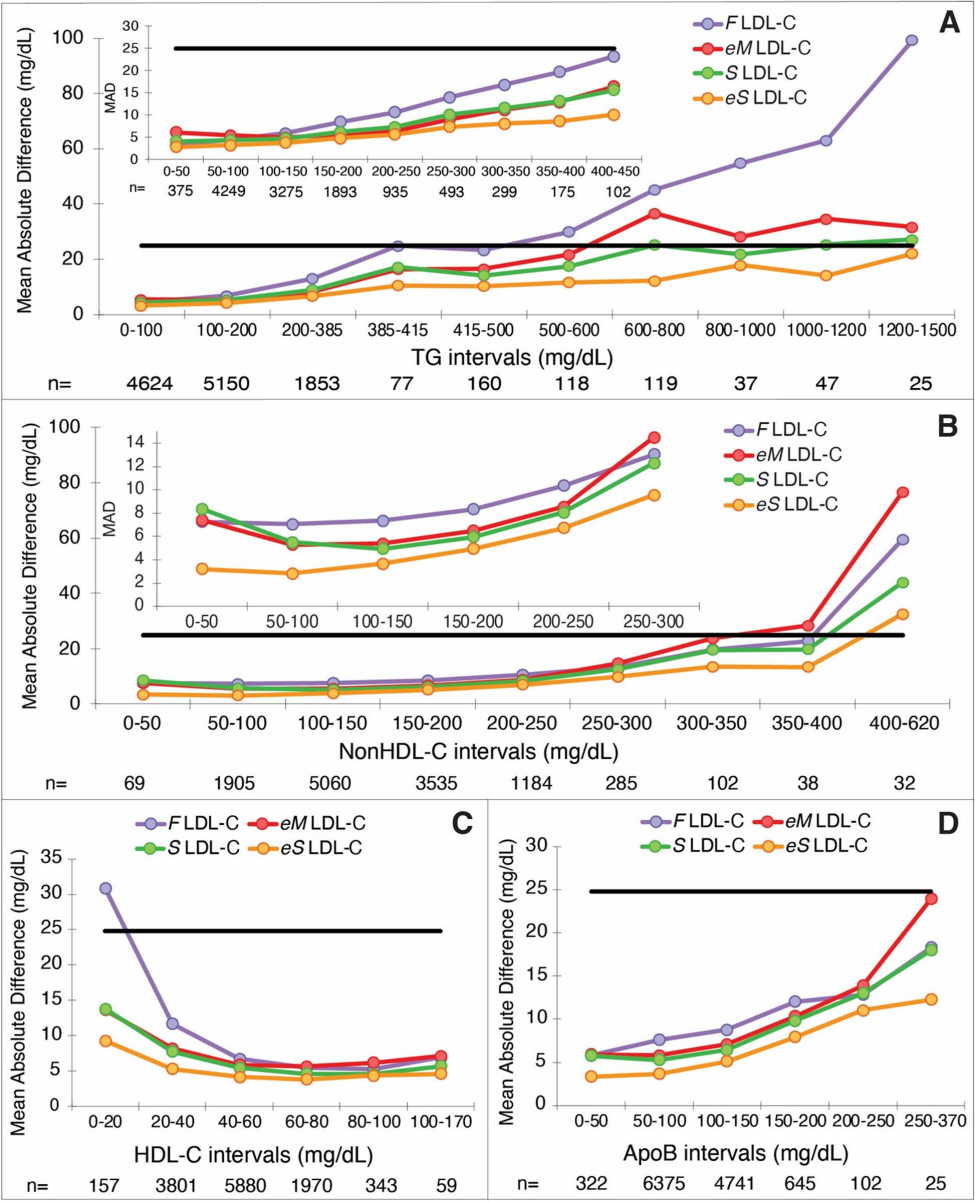
Mean absolute difference of estimated LDL-C equations at different intervals for independent variables. Mean absolute difference (MAD) of LDL-C when compared against *BQ* LDL-C from patients in the validation dataset (*N* = 12,210) is shown for the *F* LDL-C (purple line), *eM* LDL-C (red line), *S* LDL-C (green line) and *eS*-LDL-C (orange line) equations for the indicated TG intervals (**A**), nonHDL-C intervals (**B**), HDL-C intervals (**C**), and apoB intervals (**D**). The insets shows a close-up for low TG and low nonHDL-C samples. The number of samples within each interval is indicated on the X-axis. Solid black line corresponds to a MAD value of 25 mg/dL

Next, we determined with the validation dataset the MAD values for different intervals of the independent variables used in the various LDL-C equations (Fig. 3). The *F* LDL-C equation showed the largest bias compared to the other equations for hypertriglyceridemic samples, and hence the long-standing recommendation to not use this equation when TG > 400 mg/dL. The *eS* LDL-C equation maintained better accuracy as TG increased compared to other equations. Its MAD scores for TG values up to 1500 mg/dL remained below the maximum recommended error of 25 mg/dL (see solid line), which was established based on the observed error limit found for the *F* LDL-C equation at a TG of 400 mg/dL. Based on this same error limit, *S* LDL-C appears to be suitable for TG values up to 800 mg/dL as previously described [8], whereas the *eM* LDL-C equation exceeded this error limit for TG values slightly greater than 600 mg/dL. A closer examination of lower TG values (see inset) shows that the accuracy advantage of the *eS* LDL-C equation over the other equations approximately starts at TG values greater than 200 mg/dL.

Similar findings, in regard, to the superior accuracy of the eS LDL-C equation were found when the other independent variables were examined (Fig. 3). For nonHDL-C, the *eM* LDL-C equation showed the greatest bias and based on the 25 mg/dL error limit goal, it should not be used when nonHDL-C > 350 mg/dL (Fig. 3B). With respect to HDL-C, the *F* LDL-C equation showed the greatest bias when HDL-C was low and exceeded the 25 mg/dL error limit when HDL-C < 20 mg/dL (Fig. 3C). Not unexpectedly, because it is the only equation that utilizes apoB as an independent variable, the *eS* LDL-C equation showed the lowest MAD scores across a broad range of apoB values (Fig. 3D), although like the other equations its accuracy deteriorated as apoB increased.

**Fig. 4:**
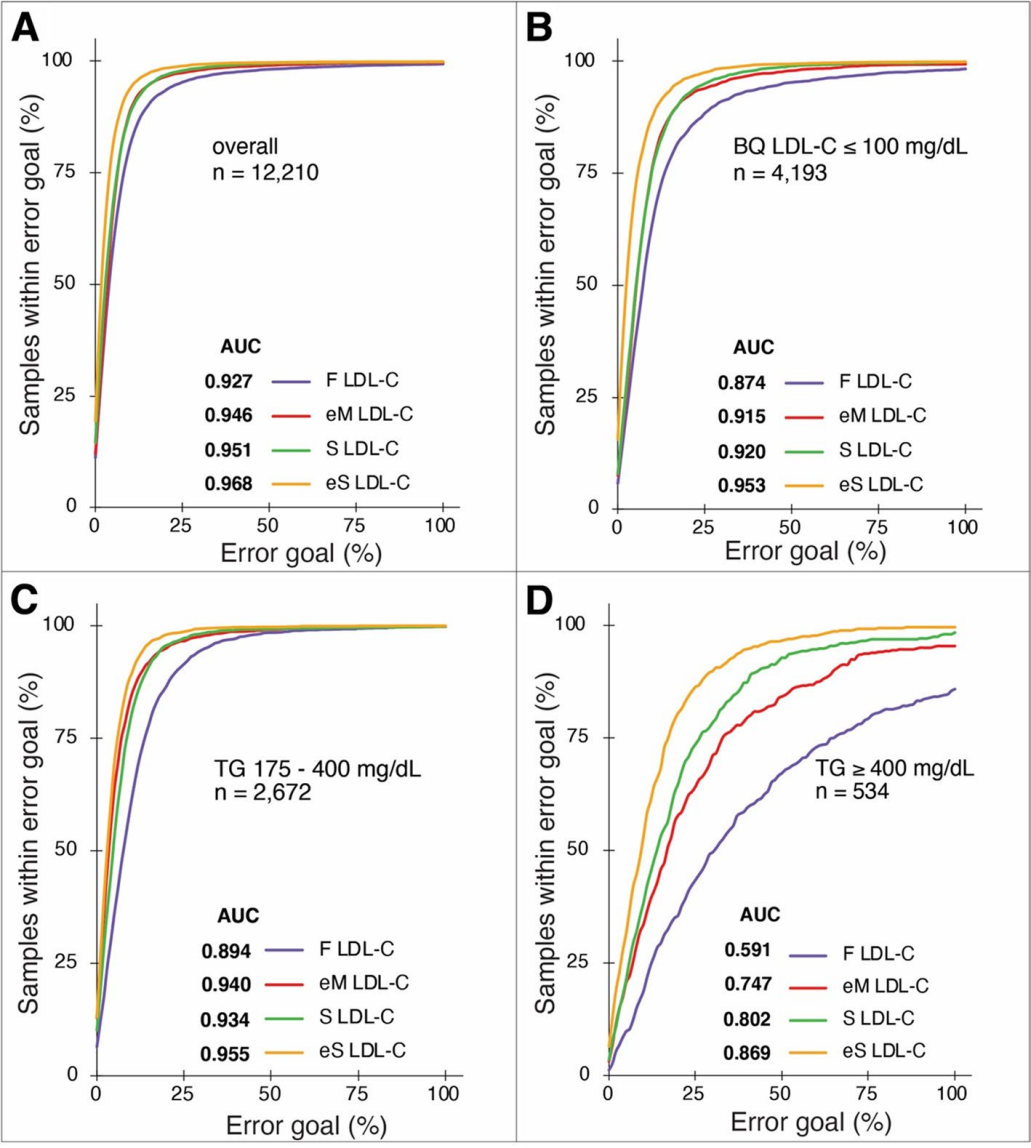
Comparison of LDL-C equations by regression error characteristic plots. Regression Error Characteristic curves for LDL-C equations, using *BQ* LDL-C as the reference method, for all the validation data (**A**), LDL-C ≤ 100 mg/dL (**B**), TG ≥ 175 mg/dL (**C**), or for TG ≥ 400 mg/dL (**D**) for *F* LDL-C (orange line), *eM* LDL-C (red line), *S* LDL-C (blue line), and *eS*-LDL-C (green line) equations. Area-under-the-curve (AUC) is calculated for each equation to provide a single integrated measure of test accuracy. Number of samples for each equation is indicated next to AUC score

In Fig. 4, we compared the accuracy of the different equations by Regression Error Characteristic analysis [20]. When the complete validation dataset was analyzed, the *eS* LDL-C equation was the most accurate and the *F* LDL-C the least accurate, which can be seen by a visual inspection of the plots or by comparing the AUC values of each equation (Fig. 4A). When we only analyzed LDL-C values below 100 mg/dL (Fig. 4B), an even greater accuracy advantage was observed for the *eS* LDL-C equation over the other equations. The *eS* LDL-C equation also provided superior accuracy when evaluating result in samples with either moderately or highly elevated triglycerides (Fig. 4C, D, respectively).

**Fig. 5:**
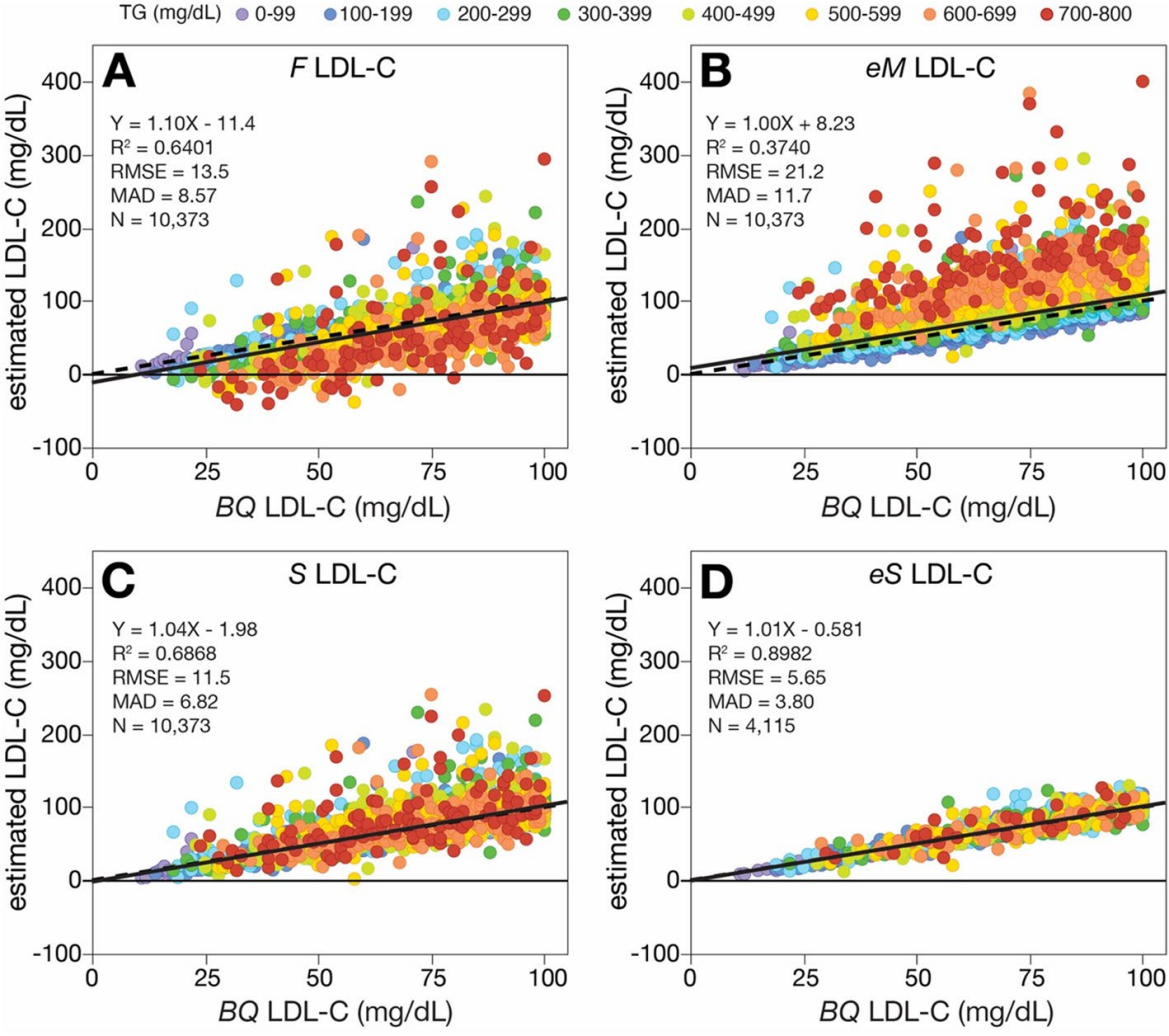
Comparison of estimated LDL-C versus *BQ*-LDL-C at low LDL-C levels. LDL-C was calculated in patients with LDL-C ≤ 100 mg/dL and TG ≤ 800 mg/dL by *F*-LDL-C (**A**, *N* = 10,373), *eM*-LDL-C (**B**, *N* = 10,373), *S-*LDL-C (**C**, *N* = 10,373), and e*S-*LDL-C (**D**, *N* = 4,115) equations and plotted against LDL-C as measured by *BQ* reference method (*BQ*-LDL-C). Solid lines are the linear fit for indicated regression equations. Dotted lines are lines of identity. Results are color coded by TG level with the values indicated in the legend (mg/dL)

Next, we compared the different equations for estimating low LDL-C values by restricting the analysis to those patients with an LDL-C < 100 mg/dL and a TG < 800 m/dL. As before when a broader set of LDL-C values were tested, the *eS* LDL-C equation had the best linear regression-based parameters of accuracy for low LDL-C samples when compared to *BQ* LDL-C (Fig. 5). Notably, the *eS* LDL-C equation had a slope of nearly 1.0 and an intercept of almost zero. A clear negative bias could be observed for the *F* LDL-C equation for high TG samples, whereas a positive bias for these same samples were observed for the *eM* LDL-C equation. This was less apparent when only samples with TG < 400 mg/dL were analyzed (Supplemental Figure 1).

**Fig. 6:**
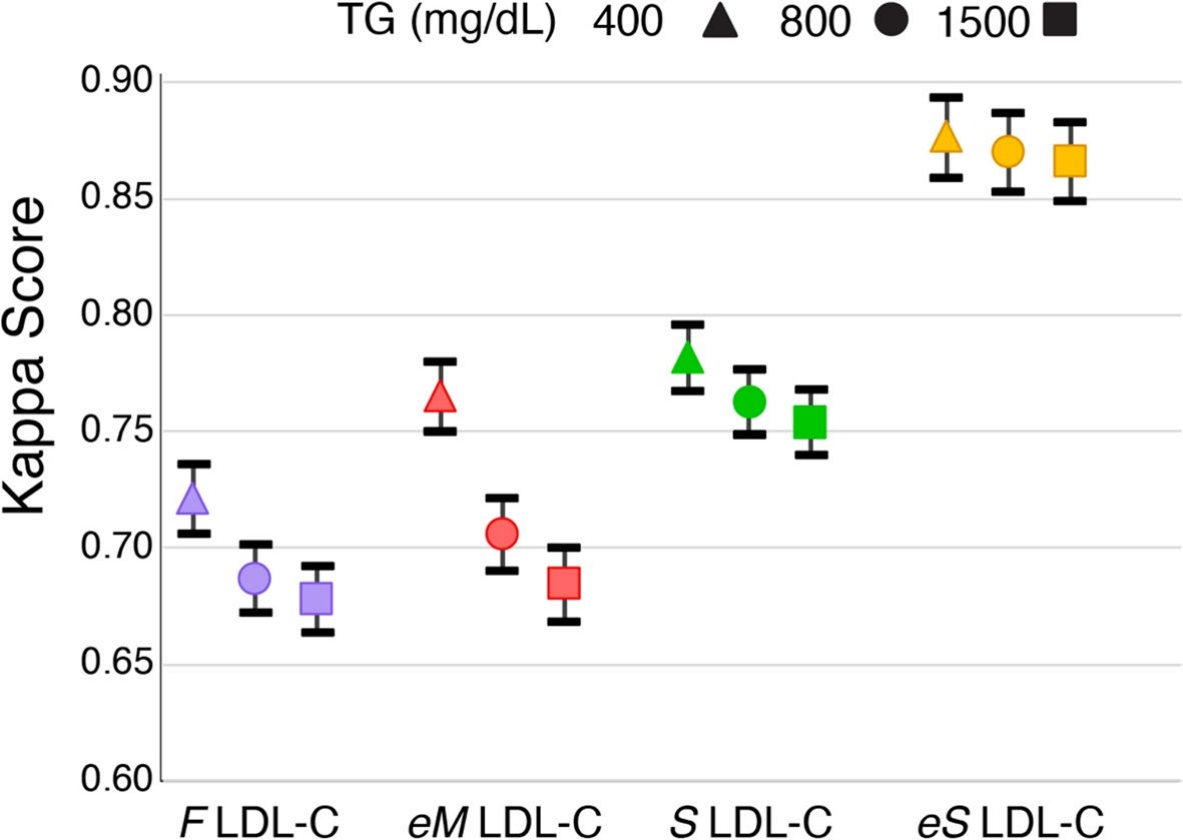
Comparison of Kappa scores of different equations for classification of patients at the 70 mg/dL cutpoint for LDL-C. Kappa score and 95% confidence intervals are shown for *F* LDL-C (purple), *eM* LDL-C (red), *S* LDL-C (green), and e*S-*LDL-C (orange) for classifying patients as below or above the 70 mg/dL LDL-C cutpoint when compared against *BQ* LDL-C for TG up to 400 mg/dL (triangles), TG up to 800 mg/dL (circles) or TG up to 1500 mg/dL (squares)

In Table 1, we tabulated the different types of classification errors by the standard LDL-C equations and the new *eS* LDL-C equation for categorizing patients as being above or below the 70 mg/dL cutpoint. True positives were defined as correctly identifying patients as being below the 70 mg/dL treatment threshold based on the *BQ* LDL-C test result. Sensitivity (for detecting patients with LDL-C < 70 mg/dL), and specificity, as well as positive predictive value (PPV) and negative predictive value (NPV) were calculated. As expected because of its negative bias, the *F* LDL-C equation showed the best sensitivity, but it had the lowest specificity. Correspondingly, it had the lowest PPV but a relatively high NPV. The *eS* LDL-C equation had the highest specificity at all three TG levels with a sensitivity almost as high as the *F* LDL-C equation. Based on the normalized Matthews Correlation Coefficient (nMCC), which combines sensitivity and specificity to obtain a single metric of accuracy [22], the *eS* LDL-C equation had the highest overall accuracy for all three TG levels, followed by the *S* LDL-C, *eM* LDL-C and *F* LDL-C equations. A similar rank order in accuracy was also found for the LDL-C equations when assessed for their agreement to the *BQ* reference method by their kappa scores (Fig. 6), another way to determine overall test accuracy [21].

**Table 1:** Classification of patients as below or above the 70 mg/dL LDL-C cutpoint by equations

Parameter	N	Sensitivity	Specificity	PPV	NPV	nMCC
TG 400 mg/dL						
Friedewald	9483	93.4	86.1	70.8	97.3	86.8
extended Martin	9483	82.0	94.1	83.4	93.5	88.2
Sampson	9483	88.7	92.1	80.2	95.7	89.2
enhanced Sampson	3894	92.6	96.1	89.4	97.3	93.8
TG 800 mg/dL						
Friedewald	10373	92.8	83.5	68.3	96.8	85.2
extended Martin	10373	73.4	94.5	83.6	90.3	85.4
Sampson	10373	86.4	91.7	80.0	94.7	88.2
enhanced Sampson	4115	92.1	95.8	89.2	97.0	93.5
TG 1500 mg/dL						
Friedewald	10611	92.8	82.6	67.7	96.7	84.8
extended Martin	10611	70.6	94.6	83.7	89.1	84.4
Sampson	10611	86.1	91.2	79.4	94.4	87.8
enhanced Sampson	4193	91.9	95.6	89.0	96.8	93.3

**Fig. 7:**
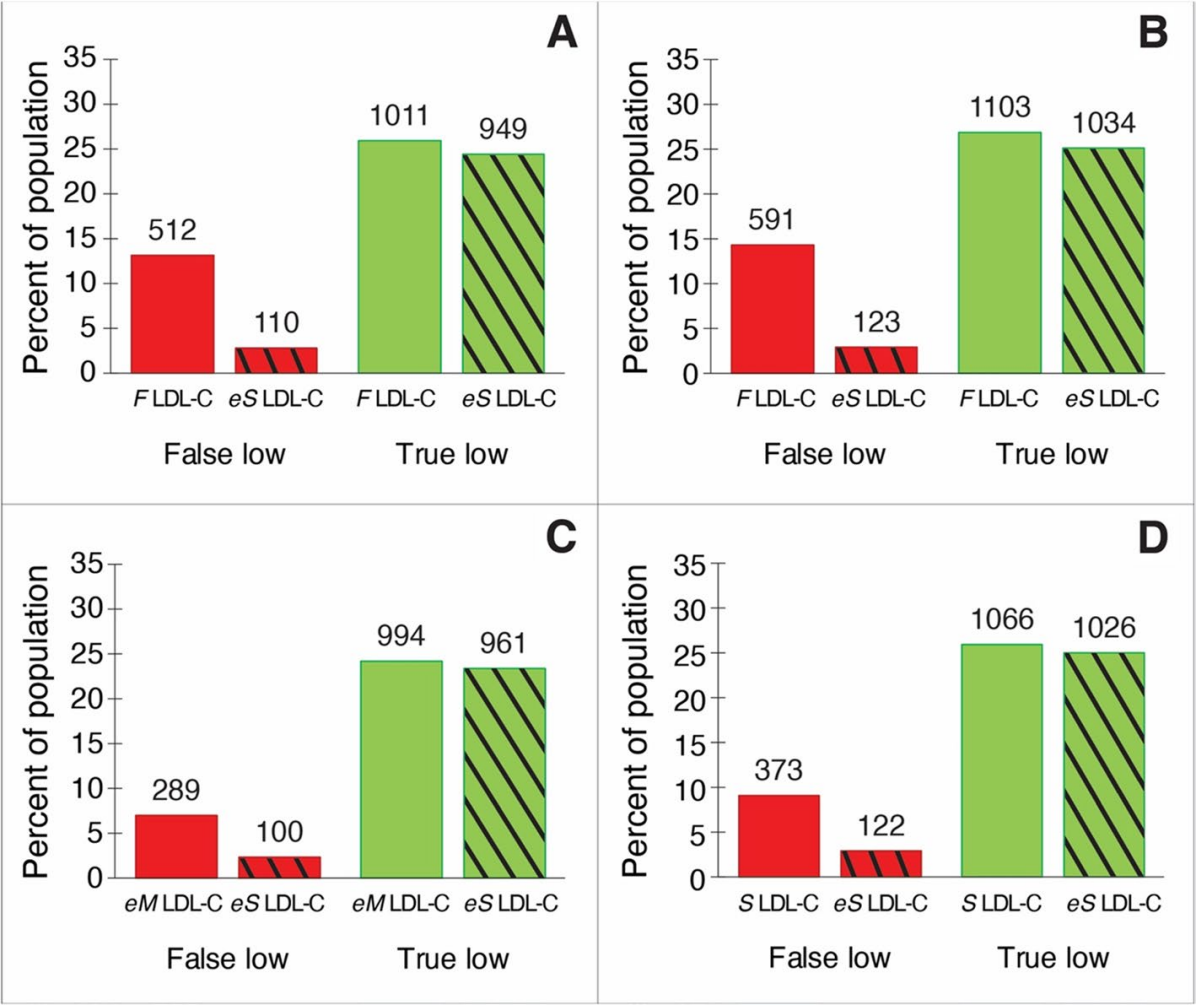
Effect of validating low estimated LDL-C results with the *eS* LDL-C equation. LDL-C was calculated by the standard equations from the validation data set that contained apoB test results. Those test results and are below the 70 mg/dL cutpoint for LDL-C are shown and classified as either being false lows or true lows by comparison against *BQ* LDL-C (red bars). False low and true low test results are also shown after repeat estimation by the *eS* LDL-C equation (red bars with black diagonal stripes). Results are shown for *F* LDL-C (**A**, TG ≥ 400 mg/dL, *N* = 3894), *F* LDL-C (**B**, TG ≥ 800 mg/dL, *N* = 4115), *eM* LDL-C (**C**, TG ≥ 800 mg/dL, *N* = 4115), and *S* LDL-C (**D**, TG ≥ 800 mg/dL, *N* = 4115) equations. Results are graphed as the percentage of the entire population with absolute numbers in each category shown over the bars

In Fig. 7, we examined the impact of first measuring LDL-C by the three currently used LDL-C equations in routine practice and then subsequently confirming the result with the *eS* LDL-C equation to simulate what might be done before deciding whether a high-risk patient is truly eligible or not for PCSK9i therapy. Based on *BQ* reference method, in about a third of patients with TG < 400 mg/dL, an LDL-C result below 70 mg/dL by the *F* LDL-C equation was falsely low (Fig. 5A). An even greater fraction of patients had falsely low test results by the *F* LDL-C equation when samples with TG up to 800 mg/dL were analyzed (Fig. 5B). When the *eS* LDL-C equation was applied to these patients, approximately 80% of the patients with falsely low results below 70 mg/dL were correctly reclassified as being higher, making them potentially eligible for PCSK9i therapy. The application of the *eS* LDL-C equation, however, resulted in a decrease in the number of truly low test results from 1011 to 949 (Fig. 5A, TG < 400 mg/dL), which could result in some high-risk patients unnecessarily receiving PCSK9i therapy. There was, however, an overall net gain of 340 patients (Fig. 5A, TG < 400 mg/dL) that were correctly identified as being eligible for PCSK9i therapy by the *eS* LDL-C equation. Similarly, the use of the *eS* LDL-C equation as a confirmatory test also decreased the number of falsely low results when applied to the *M* LDL-C (Fig. 6C) and *S* LDL-C (Fig. 6D) equations for TG values up to 800 mg/dL, but to a lesser degree than for the *F* LDL-C equation, because these newer equations had less falsely low results to begin with. Like for the *F* LDL-C equation, using the *eS* LDL-C equation as a confirmatory test resulted in net gain of correctly classified patients for these other two equations as well. Consistent with its higher PPV but lower NPV (Table 1), the *eM* LDL-C equation had a lower number of false low test results than the *S* LDL-C equation but also a lower number of true low test results.

## Discussion

In this study, we describe the development and validation of a new equation for LDL-C that includes apoB as an independent variable. The new *eS* LDL-C equation outperforms, in terms of accuracy, all the other commonly used equations for calculating LDL-C. It is suitable for samples with TG values up 1500 mg/dL, which is much higher than the other equations. It also had the best performance in patients with low LDL-C.

In the United States, nearly 9 million adults with ASCVD fail to achieve optimal LDL-C levels, despite the use of maximally tolerated statin therapy [23]. It is currently recommended that high-risk patients that do not attain an LDL-C value below 70 mg/dL be treated with an additional lipid-lowering drugs, such as PCSK9i therapy [1]. When it was first approved by the FDA, as many as half to three quarters of all eligible patients were initially denied insurance coverage for PCSK9i therapy [24]. Although the current reimbursement situation is much improved, high-risk patients with a falsely low LDL-C below 70 mg/dL are still not likely to receive this relatively expensive treatment if the current guidelines and eligibility criteria for reimbursement are strictly followed. Due to the recognized limitations of the *F* LDL-C equation, particularly its negative bias in hypertriglyceridemia patients, the US-Multi-Society cholesterol guidelines recommended in 2018 [1] the use of either a direct LDL-C test or the *M* LDL-C equation for low LDL-C values to mitigate this problem.

In 2020, the *S* LDL-C equation was developed and like the *M* LDL-C equation, it is more accurate than the *F* LDL-C equation, particularly for patients with hypertriglyceridemia [8, 9]. It differs from the *M* LDL-C equation in that it was developed using the *BQ* reference method, a swinging bucket ultracentrifuge procedure that also includes an LDL precipitation step. All routine diagnostic assays for LDL-C are standardized against this reference method by the Centers for Disease Control and Prevention (CDC). It should be noted that the *BQ* reference method for LDL-C can sometimes include cholesterol from Lp(a) and from some denser remnant particles, but these lipoprotein subfractions are also believed to be proatherogenic. The *M* LDL-C equation used the VAP method as its reference method, a rapid ultracentrifugation method that can result in the under recovery of VLDL-C on hypertriglyceridemic samples [6, 13], leading to an overestimation of LDL-C. When compared against the *BQ* reference method, the *S* LDL-C equation is slightly more accurate than the original Martin or *eM* LDL-C equations [8–10]. It still, however, has less than ideal accuracy for low LDL-C samples [9], which prompted us to develop the new *eS* LDL-C equation.

Not unexpectedly, the inclusion of apoB as an independent variable in the *eS* LDL-C equation substantially improved its accuracy. It likely does so by providing the particle count of all apoB-containing lipoproteins. Although, this includes not only LDL but also VLDL particles (or remnants), the great majority of apoB is on LDL for most patients. Thus, inclusion of apoB likely improved the prediction of LDL-C by providing information related to the number of LDL particles present. ApoB, however, does not provide information related to the size of lipoprotein particles, another important determinant of the cholesterol carrying capacity of lipoproteins, but this information is provided, at least in part, by the total TG level, which is used in the new equation. When TG are greatly elevated as in patients with Type I hyperlipidemia [25], very large size VLDL particles or chylomicrons (in non-fasting samples) are markedly increased from deficient lipolysis, but because of their high TG carrying capacity, the concentration of apoB may not be correspondingly increased [11]. In contrast, apoB is typically elevated in patients with moderate hypertriglyceridemia, because of the increased number of small dense LDL particles in these patients due to CETP-mediated lipid exchange and the subsequent increased lipolysis of LDL [26]. LDL-C, however, is often normal or even decreased as measured by the *BQ* reference method or by other methods in patients with moderate hypertriglyceridemia. This is because large size LDL particles, which are inversely related to the TG level, typically account for the majority of the cholesterol that is transported on LDL. Thus, the use of apoB for estimating LDL-C adjusts for this complex relationship between TG and LDL-C and improves the accuracy of the *eS* LDL-C equation.

A major limitation of our new equation is that it involves additional laboratory testing, namely the measurement of apoB, and hence increases the cost for estimating LDL-C compared to the other equations. If used, however, as described in this study to only confirm low LDL-C values below 70 mg/dL on high-risk patients being considered for adding new lipid-lowering therapy, it would not increase overall costs too much because of the relatively low number of these type of patients. It is also worth noting that many studies have now shown that apoB and non-HDL-C are superior to LDL-C for ASCVD prediction and monitoring [27, 28]. Furthermore, treatment to apoB target goals, which typically involves more aggressive lipid-lowering therapy, reduces ASCVD events to a greater extent than treatment goals based on LDL-C [10, 21, 28, 29]. Eventually, LDL-C should possibly be replaced with apoB or another more predictive biomarker, but in the meantime until guidelines change and insurance companies change their reimbursement policies, using apoB in the *eS* LDL-C equation for reducing the number of patients with falsely low LDL-C can be a useful interim approach. It is also worth noting that the cost of apoB testing is relatively trivial (typically under 50 US dollars) compared to the cost of PCSK9i therapy, which typically costs several thousand dollars a year and are usually recommended for the life of a patient [30, 31].

## Conclusions

The *eS* LDL-C equation, which utilizes apoB as an independent variable, is the most accurate method for estimating LDL-C. When used to confirm low LDL-C values that were first determined by any of the three commonly used LDL-C equations in routine practice, it can reduce the number of high-risk patients with falsely low LDL-C results, who may not otherwise be treated with the new more effective and potentially life-saving lipid-lowering therapies. Furthermore, the more accurate measurement of LDL-C with the use of apoB should improve the adherence to current guidelines for using PCSK9i therapy based on LDL-C values, and should, therefore, be cost effective [30] and reduce ASCVD events, which costs the healthcare system in the US between 30–40 billion dollars a year.

### Supplementary Information

**Additional file 1: Supplemental Figure 1.** Comparison of estimated LDL-C versus *BQ*-LDL-C at low levels. LDL-C was calculated in patients with LDL-C ≤ 100 mg/dL and TG ≤ 400 mg/dL by F-LDL-C (Panel A, N=9,483), eM-LDL-C (Panel B, N=9,483), S-LDL-C (Panel C, N=9,483), and eS-LDL-C (Panel D, N=3,894) equations and plotted against LDL-C as measured by BQ reference method (BQ-LDL-C). Solid lines are the linear fit for indicated regression equations. Dotted lines are lines of identity. Results are color coded by TG level with the values indicated in the legend (mg/dL).

## Data Availability

Data for key findings and a spreadsheet for performing the new *eS* LDL-C calculation can be downloaded at the NHLBI Fig Share website by searching under the name Sampson.

## Abbreviations

LDL-C: Low-density lipoprotein cholesterol
PCSK9: Proprotein convertase subtilisin/kexin type 9
PCSK9i: PCSK9 inhibitors
eS LDL-C: Enhanced Sampson-NIH equation
BQ: β- Quantification reference method
S LDL-C: Sampson-NIH equation
F LDL-C: Friedewald equation
M LDL-C: Martin-Hopkins equation
eM LDL-C: Extended Martin-Hopkins equation
ASCVD: Atherosclerotic Cardiovascular Disease
TC: Total cholesterol
HDL-C: High-density lipoprotein-cholesterol
TG: Triglycerides
VLDL: Very low-density lipoproteins
VAP: Vertical Auto Profile
apoB: Apolipoprotein B
