# Supplementary figures and images for: An improved method for estimating low LDL-C based on the enhanced Sampson-NIH equation
Source: Lipids Health Dis. 2024 Feb 8;23:43. doi: 10.1186/s12944-024-02018-y (PMC10851542; doi:10.1186/s12944-024-02018-y)

**Supplemental figure 1**

**
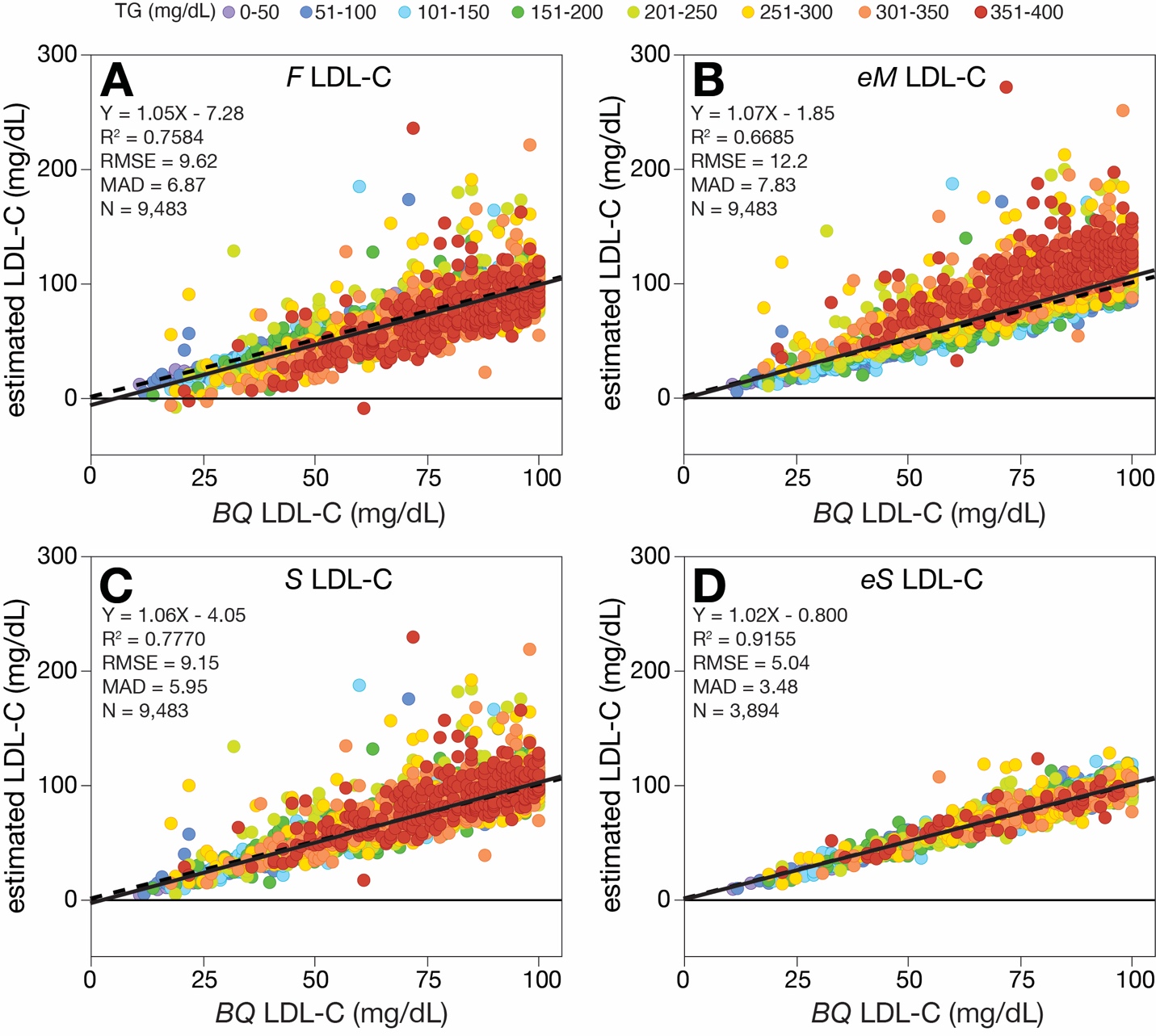
**

Supplement: Supplementary file 1 — Additional file 1: Supplemental Figure 1. Comparison of estimated LDL-C versus BQ-LDL-C at low levels. LDL-C was calculated in patients with LDL-C ≤ 100 mg/dL and TG ≤ 400 mg/dL by F-LDL-C (Panel A, N=9,483), eM-LDL-C (Panel B, N=9,483), S-LDL-C (Panel C, N=9,483), and eS-LDL-C (Panel D, N=3,894) equations and plotted against LDL-C as measured by BQ reference method (BQ-LDL-C). Solid lines are the linear fit for indicated regression equations. Dotted lines are lines of identity. Results are color coded by TG level with the values indicated in the legend (mg/dL). [file 12944_2024_2018_MOESM1_ESM.docx]
